# End-of-life decision-making and changing preferences in patients with a left ventricular assist device for destination therapy: insights from advance directives in Japan

**DOI:** 10.1007/s10047-025-01519-6

**Published:** 2025-07-22

**Authors:** Toshihide Izumida, Eisuke Amiya, Masaru Hatano, Junichi Ishida, Miki Kanno, Asako Shimada, Miyoko Endo, Masahiko Ando, Mitsutoshi Kimura, Shogo Shimada, Minoru Ono, Norihiko Takeda

**Affiliations:** 1 Department of Cardiovascular Medicine, Graduate School of Medicine, The University of Tokyo, Bunkyo-ku, Tokyo, 113-8655 Japan; 2 Department of Organ Transplantation, The University of Tokyo Hospital, Tokyo, Japan; 3 Department of Nursing, The University of Tokyo Hospital, Tokyo, Japan; 4 Department of Cardiac Surgery, Graduate School of Medicine, The University of Tokyo, Tokyo, Japan

**Keywords:** Advance directive, Advanced care planning, Durable left ventricular assist device, Palliative care, Quality of life

## Abstract

End-of-life decision-making and the evolution of patient preferences over time remain insufficiently explored in destination therapy with left ventricular assist device (DT-LVAD) in Japan. This retrospective observational study analyzed standardized advance directives from DT-LVAD patients in Japan. The advance directives comprised (1) preferred end-of-life medical care, (2) designated surrogate decision-makers, and (3) personal life wishes. This study comprises two components: a cross-sectional analysis of all patients at the time of LVAD implantation, and a longitudinal analysis evaluating changes in advance directive preferences among patients who completed the 1-year follow-up assessment. This study included 27 patients who initially received DT-LVAD (median age 47 years, 21 men). At the time of LVAD implantation, approximately 30% of DT-LVAD patients preferred continued mechanical support, including LVAD, mechanical ventilator, and hemodialysis, until the end-of-life stage in a cross-sectional analysis. In addition, 56% preferred receiving end-of-life care at home. In a longitudinal analysis, patients remaining on DT-LVAD showed increased preference for “independence” and “end-of-life care at home” after 1 year of DT-LVAD, whereas their inclination toward invasive treatments decreased. In contrast, patients switched to BTT demonstrated lower inclination toward invasive therapies from the outset, with this tendency becoming more pronounced over time. This study demonstrates the changes in the end-of-life preferences of DT-LVAD patients in Japan. With the continuous increase in the number of DT-LVAD patients, further refinement of advance directive frameworks and the development of structured community-based support systems will be essential for optimizing end-of-life care among LVAD recipients.

**Supplementary Information:**

The online version contains supplementary material available at 10.1007/s10047-025-01519-6.

## Introduction

Destination therapy with left ventricular assist device (DT-LVAD) was introduced in Japan in 2021, which led to the expansion of treatment options for patients with advanced heart failure [1]. As of 2024, 169 DT-LVAD cases had been recorded nationwide, and this number has been continuously increasing [2]. Unlike bridge to transplantation (BTT), DT-LVAD is unique in that all patients inevitably reach the end-of-life, making comprehensive patient and family care, including end-of-life support, a critical priority [3].

A distinctive feature of DT-LVAD in Japan is the mandatory completion of an advance directive for all patients along with an annual follow-up using a standardized form across all facilities [1]. Unlike conventional advance directives, which mainly outline preferred medical treatments and designate surrogate decision-makers at the end-of-life stage, this form includes each patient’s life wish, providing insights into their values and priorities. While the clinical impact of traditional advance directives remains inconclusive, no study has specifically investigated the importance of this unique approach in DT-LVAD [4]. Considering that DT-LVAD in Japan involves structured, multidisciplinary, and multicenter discussions with repeated advance care planning (ACP), this framework may have distinct value warranting further investigation [1].

To elucidate the current state of end-of-life preferences and advance directives in DT-LVAD in Japan, we conducted a descriptive observational study to examine (1) the characteristics of advance directives at the time of DT-LVAD implantation and (2) longitudinal changes in the preferred medical treatments and life wishes over time among patients receiving LVAD therapy as DT.

## Materials and methods

### Patient selection

This study included consecutive patients aged ≥ 18 years who received DT-LVAD at our facility from April 2021 to December 2024 and had a documented advance directive. Our hospital is one of the 11 heart transplant centers in Japan and a leading tertiary care institution with extensive experience in heart transplant. Patients with advanced heart failure are referred to our hospital from neighboring prefectures within 200 km.

This study was conducted in accordance with the principles of the Declaration of Helsinki and approved by the Ethics Committee of the Faculty of Medicine at the University of Tokyo (approval number: 2650). The requirement for informed consent was waived due to the retrospective nature of the study.

### Destination therapy in Japan

The Japan Circulatory Society guideline and consensus report define the detailed criteria for DT-LVAD indication in Japan [1, 5]. In summary, DT is indicated for patients with advanced heart failure who are ineligible for heart transplantation, including those aged ≥ 65 years and those aged < 65 years who do not fully qualify for transplantation due to several factors, such as occult cancer. As of December 2024, there were 20 DT-LVAD facilities in Japan. The treatment indications are reviewed by an Indication Review Committee established at each institution, and the results need to be submitted to the Council for Clinical Use of Ventricular Assist Device Related Academic Societies. HeartMate 3 (Abbott, Abbott Park, Illinois, USA) is the only DT-LVAD model approved and used in all cases in Japan [1].

### Advance directive for DT-LVAD in Japan and the descriptive method

The advance directives for DT-LVAD in Japan were comprehensively examined in a previous report [1]. These directives mainly comprise (1) preferred end-of-life medical care, (2) designated surrogate decision-maker, and (3) personal life wishes. The directives include 10 specific end-of-life medical interventions, for which patients indicate their preference by selecting “I prefer,” “I don’t prefer,” or “I don’t know” [1]. The medical interventions are as follows: (1) sustained support by an implantable LVAD, (2) use of an intravenous inotrope, (3) blood transfusion, (4) mechanical ventilation, (5) artificial heart–lung machine, (6) chest compression for resuscitation, (7) hemodialysis, (8) gastrostomy tube feeding, (9) nasogastric tube feeding, and (10) intravenous feeding. For the purpose of this study, the responses “I don’t prefer” and “I don’t know” were categorized together as “other.” In addition, the directive captures six main personal life wishes: (1) spend enough time with my loved one, (2) live without other’s help as possible, (3) eat and use the toilet by myself, (4) avoid showing weakness to others, (5) live in a quiet space, and (6) be treated by all medical measures as far as possible to recover.

The timing of advance directive acquisition may vary depending on the patient’s clinical status. For those with an interagency registry for mechanically assisted circulatory support (INTERMACS) profile of 3 or higher, the directives were obtained preoperatively. In contrast, for those with an INTERMACS profile of 2 or those unable to complete directives in the acute phase, documentation was finalized postoperatively following the LVAD implantation. The advance directives were followed up at least annually and reassessed as needed after significant clinical events.

### Data analysis

Simple descriptive analyses were conducted using Excel 2016 and SPSS Statistics 22 (IBM, Chicago, IL, USA). Continuous variables were expressed as median and interquartile range, whereas categorical variables were expressed as numbers and percentages. No group comparisons were made; thus, inferential statistics were not performed.

This study comprises two components: (1) a cross-sectional analysis of all patients at the time of LVAD implantation, and (2) a longitudinal analysis evaluating changes in advance directive preferences among patients who completed the 1-year follow-up assessment. To evaluate longitudinal changes in advance directive preferences, analyses were limited to patients who completed follow-up of advance directive at 1 year post-implantation. Patients who died, transferred to another facility, or were lost to follow-up were excluded from this analysis to ensure consistent denominators and accurate proportions.

## Results

### Cross-sectional analysis

#### Baseline characteristics

**Fig. 1:**
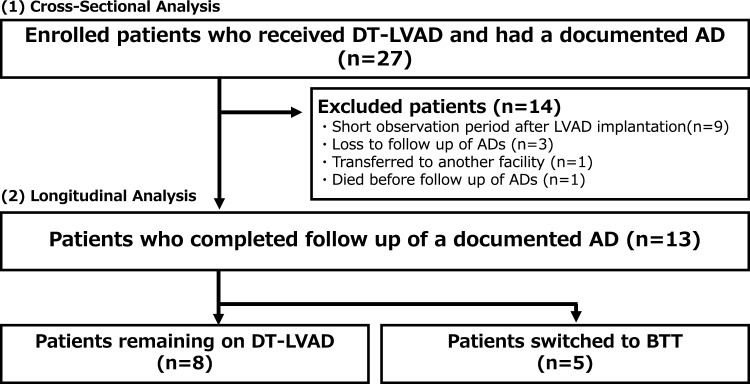
Flowchart. AD, advance directive; BTT, bridge to transplantation; DT, destination therapy; LVAD, left ventricular assist device

This study included 27 patients (median age 47 [37, 53] years, 21 men) in Fig. 1. As shown in Table 1, the median number of heart failure-related hospitalizations was 3 (1, 7), and the median duration from the first heart failure hospitalization to LVAD implantation was 3.2 (0.2, 13.9) years. The spouse served as the surrogate decision-maker in 23 cases (85%).

**Table 1:** Baseline data

	All (*n* = 27)	Longitudinal follow-up cases	
		Remaining on DT-LVAD (*n* = 8)	Switching to BTT (*n* = 5)
Age, years	57 (46, 64)	63 (53, 68)	56 (41, 57)
Male, *n* (%)	21 (78%)	5 (63%)	4 (80%)
Causes of cardiac disease, *n* (%)			
Dilated cardiomyopathy	12 (44%)	4 (50%)	1 (20%)
Ischemic cardiomyopathy	9 (33%)	2 (25%)	2 (40%)
CRT/ICD, *n* (%)	13 (48%)	6 (75%)	2 (40%)
Numbers of hospitalization for heart failure, *n*	3 (1, 7)	6 (2, 9)	1 (1, 4)
Duration from the first heart failure hospitalization, years	3.2 (0.2, 13.9)	9.3 (3.9, 15.2)	0.3 (0.1, 20.9)
Surrogate decision-maker, *n* (%)			
Spouse	23 (85%)	5 (63%)	3 (60%)
Parents	2 (7%)	1 (12%)	1 (20%)
Siblings	2 (7%)	1 (12%)	1 (20%)
Children	1 (4%)	1 (12%)	0 (0%)
INTERMACS profile, *n* (%)			
2	13 (48%)	3 (38%)	3 (60%)
3	11 (41%)	3 (38%)	2 (40%)
4	3 (11%)	2 (25%)	0 (0%)
Preoperative use of temporary MCS, *n* (%)	10 (37%)	3 (38%)	3 (60%)
Intra-aortic balloon pump	3 (11%)	2 (25%)	1 (20%)
Percutaneous transvalvular microaxial flow pump	7 (26%)	1 (13%)	2 (40%)
Venoarterial extracorporeal membrane oxygenation	0 (0%)	0 (0%)	0 (0%)
Preoperative dialysis, *n* (%)	0 (0%)	0 (0%)	0 (0%)
History of mechanical ventilation^a^, *n* (%)	7 (26%)	1 (13%)	2 (40%)

Continuous variables were expressed as median and interquartile range, while categorical variables were expressed as numbers and percentages

BTT, bridge to transplant; CRT, cardiac resynchronization therapy; DT, destination therapy; ICD, implantable cardioverter defibrillator; INTERMACS, interagency registry for mechanically assisted circulatory support; LVAD, left ventricular assist device; MCS, mechanical circulatory support

^a^“History of mechanical ventilation” refers to a history of invasive mechanical ventilation, not its use at the time of LVAD implantation

#### End-of-life medical preferences and life wishes at the perioperative phase in patients undergoing LVAD implantation

**Fig. 2:**
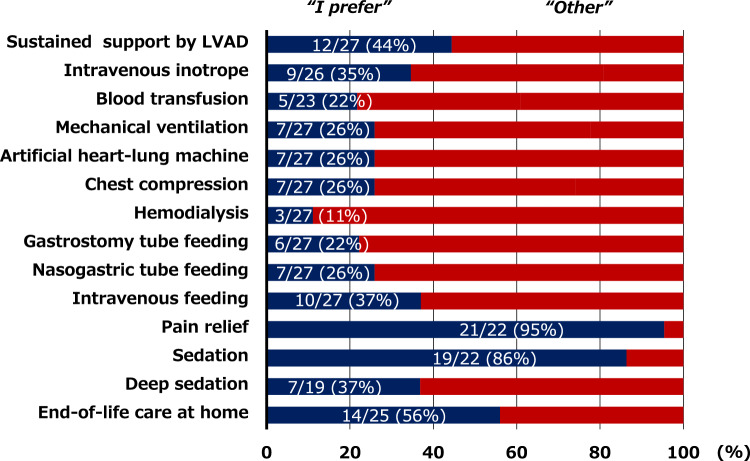
End-of-life medical preferences at the time of LVAD implantation in patients undergoing destination therapy. LVAD, left ventricular assist device

**Fig. 3:**
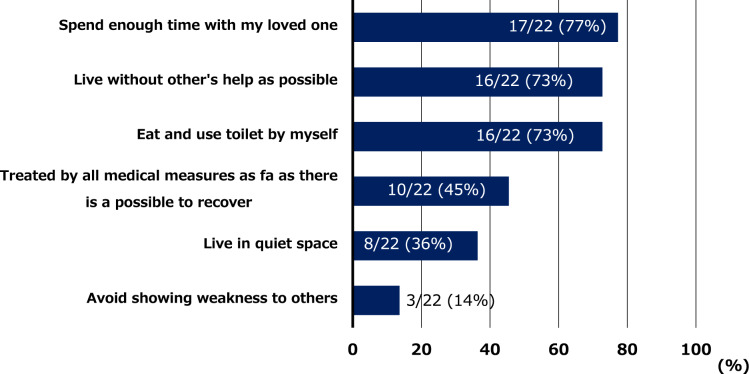
Life wishes at the time of LVAD implantation in patients undergoing destination therapy. LVAD, left ventricular assist device

As shown in Fig. 2, approximately 30% of patients who initially received DT-LVAD wished to continue LVAD support, mechanical ventilation, and other invasive treatments at the end-of-life stage. In addition, 56% of the DT-LVAD patients preferred receiving end-of-life care at home. As shown in Fig. 3, the most common wish among the DT-LVAD patients was to “spend time with loved ones” (77%), followed by “independence in daily life” (73%).

### Longitudinal analysis

#### Changes in the end-of-life preferences and life wishes of patients remaining on DT-LVAD and those switched to bridge to transplantation

As shown in Fig. 1, 13 of the 27 enrolled patients completed both baseline and 1 year follow-up assessments of advance directives. Among them, 8 patients remained on DT-LVAD, while 5 were switched to BTT. All patients who transitioned to BTT were initially managed under a “bridge to candidacy” strategy. Specifically, three patients were initially designated as DT due to hemodynamic instability that precluded completion of the required transplant evaluation tests, and two others had uncertain reversibility of end-organ dysfunction, particularly renal impairment. These conditions prevented immediate transplant listing; however, following clinical stabilization after LVAD implantation, all five patients underwent reassessment and were subsequently deemed eligible for transplant. These transitions occurred within the 1 year following LVAD implantation and prior to the 1 year follow-up assessment.

The baseline characteristics of these two groups are summarized in Table 1. The median age was 63 (53, 68) years for patients remaining on DT-LVAD and 56 (41, 57) years for those switched to BTT. The median number of heart failure-related hospitalizations before LVAD implantation was 6 (2, 9) in the former group and 1 (1, 4) in the latter.

**Fig. 4:**
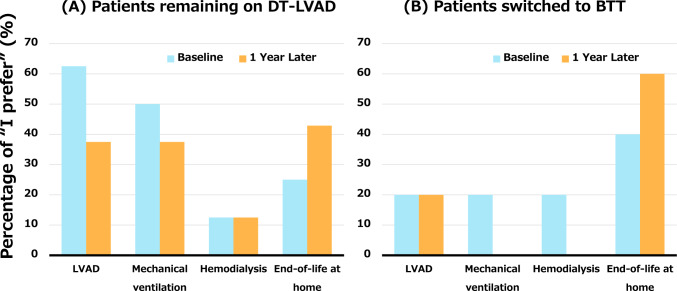
End-of-life medical preferences at the time of LVAD implantation and 1 year post-implantation in **A** patients remaining on DT-LVAD and **B** those switched to BTT. BTT, bridge to transplantation; DT, destination therapy; LVAD, left ventricular assist device

As shown in Fig. 4A and Supplementary Fig. 1A and 2A, the proportion of patients remaining on DT-LVAD who prioritized “end-of-life care at home” increased after 1 year of LVAD therapy, whereas the proportion of those who preferred “continuation of LVAD therapy,” “mechanical ventilation,” and “dialysis” decreased. Meanwhile, few patients switched to BTT initially preferred invasive treatments at the time of LVAD implantation, and this tendency became even more pronounced over time, as shown in Fig. 4B and Supplementary Fig. 1B and 2B.

**Fig. 5:**
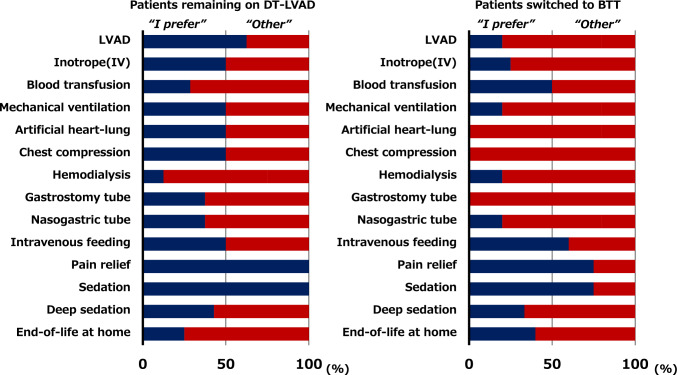
End-of-life medical preferences at the time of LVAD implantation in patients remaining on destination therapy and those switched to bridge to transplantation. BTT, bridge to transplantation; DT, destination therapy; IV, intravenous; LVAD, left ventricular assist device

As depicted in Fig. 5, the patients remaining on DT-LVAD were more likely to request invasive interventions, including “continuation of LVAD therapy,” “mechanical ventilation,” and “dialysis,” than those switched to BTT.

#### Advance directives and end-of-life care in the deceased patient

Among the study cohort, one patient died during the follow-up period. This 65-year-old man, who received pure DT-LVAD therapy, had clearly documented a wish in his advance directive to avoid aggressive treatments and prioritized comfort-focused care in a quiet environment in a quiet setting with loved ones. In accordance with these stated wishes, he remained at home, surrounded by family, and passed away peacefully.

## Discussion

Our study demonstrated several key findings: (1) According to the advance directive, the most common life wish among the DT-LVAD patients was to “spend time with loved ones,” followed by “independence in daily life.” Notably, more than 50% of patients preferred to receive end-of-life care at home. (2) In terms of end-of-life care preferences, over 30% of patients desired to receive active treatments, such as continuation of LVAD therapy, mechanical ventilation, and hemodialysis, whereas the proportion of patients opting against such interventions increased over time. (3) There may be differences in end-of-life care preferences between patients remaining on DT-LVAD and those switched from DT to BTT.

### Clinical implications and future directions of advance directives for DT-LVAD in Japan

This is the first study to examine the current state of advance directives for DT-LVAD in Japan, focusing on patients’ preferences and how their end-of-life care wishes evolve over time.

The constantly progressive nature of heart failure, characterized by recurrent acute exacerbations, may inevitably promote ACP, which includes shared decision-making among patients, families, and healthcare professionals [6]. This process is further reinforced by multidisciplinary involvement, potentially enhancing satisfaction with end-of-life care for the patients and their families [7, 8]. One key advantage of standardization for advance directive is its ability to promote clear and consistent communication of patients’ wishes [1].

However, the experience with end-of-life care in DT-LVAD patients in Japan remains relatively limited, and the actual clinical impact of advance directive remains unknown. Notably, one patient in this cohort died during follow-up and was able to spend his final days at home in accordance with the preferences outlined in his advance directive. This case illustrates the potential utility of advance directives in aligning end-of-life care with patient values. While the current standardized format is clear and easy to use, it may not fully capture the depth of each patient’s values. A more comprehensive assessment—including factors, such as what brings them joy, what makes life meaningful, and their primary concerns—could further enhance the ACP process. Future revisions to the advance directive framework should consider the integration of these elements to better reflect individual patient priorities.

### Palliative care and end-of-life decision-making in DT-LVAD and BTT-LVAD patients

End-of-life care preferences appear to differ between patients remaining on DT-LVAD and those transitioned to BTT, with DT-LVAD patients more frequently favoring aggressive treatment even at the terminal stage.

The reasons for these differences remain unclear but may be associated with clinical and psychological factors such as pre-implant status, ambiguity around transplant eligibility in “bridge to candidacy” cases, and differences in therapeutic goals between DT and BTT strategies [9, 10]. Notably, in our cohort, patients who were ultimately designated as BTT had a higher incidence of intensive pre-implantation interventions such as temporary mechanical circulatory support. Although these variables were not the direct focus of our interviews, such interventions may contribute to heightened psychological vulnerability, which could in turn shape patients’ subsequent attitudes and preferences regarding end-of-life care.

Although this study was not designed to explore causality, our findings highlight the need to assess end-of-life preferences separately for DT-LVAD and BTT-LVAD patients. Future research with larger cohorts and longitudinal evaluations of clinical trajectories, psychosocial factors, and evolving treatment goals are warranted to better elucidate the underlying mechanisms and inform tailored approaches to ACP in these patient groups.

### Direction and needs for end-of-life care in DT-LVAD in Japan

Despite the fact that more than half of DT-LVAD patients in clinical practice wish to spend time with their loved ones and receive end-of-life care at home, a supportive social infrastructure for adequate LVAD management remains lacking [11]. To enhance end-of-life care for these patients, it will be necessary to further refine heart failure teams at each facility and implement various initiatives, such as training programs for LVAD supporters, awareness campaigns for emergency teams and local residents, expansion of shared care networks between VAD implantation centers and management facilities, and educational efforts to strengthen community-based care.

This observational study reveals a noteworthy shift in patient preferences, with an increasing number of DT-LVAD patients in Japan who do not wish to continue LVAD support as they approach the end of life. As a result, the need for comprehensive discussions on LVAD deactivation is expected to increase, making it crucial to develop clear decision-making frameworks for end-of-life care in DT-LVAD patients [1]. Considering the complexity of LVAD deactivation, careful decision-making must take place within a highly intricate ethical and moral framework, requiring multidisciplinary evaluation and judgment that align with each patient’s values and preferences [12].

### Limitations

This study has several limitations. First, as a proof-of-concept study, the sample size was small and the follow-up duration was relatively short. However, given the anticipated increase in the number of DT-LVAD patients, understanding their end-of-life medical needs in advance is crucial. Second, further investigation is warranted to determine whether the implementation of advance directives can effectively improve patients’ quality of life. Third, the impact of post-LVAD hospitalization events on advance directives remains unclear. In this study, few patients were hospitalized after LVAD implantation. Further analysis is necessary as more cases accumulate. Fourth, the generalizability of this descriptive study is limited by potential population representativeness. However, our facility carries out approximately 20% of all DT-LVAD implantations in Japan, which strengthens the relevance of our findings [2]. Fifth, our study did not include a cross-cultural comparison of end-of-life care preferences in LVAD patients. While cultural context plays a significant role in shaping such decisions, comparative data across countries remain limited. Future multinational studies using standardized tools are warranted to better understand cultural influences in this domain.

## Conclusion

This study highlights the dynamic nature of end-of-life preferences among DT-LVAD patients in Japan. Over time, a shift was observed from preference for continued mechanical support toward greater emphasis on independence and home-based end-of-life care. As the number of DT-LVAD patients continues to increase, further refinement of advance directive frameworks will be essential to ensure that patient preferences are appropriately reflected in clinical decision-making. In addition, establishing structured, community-based support systems will be critical in optimizing end-of-life care for LVAD recipients.

## Supplementary Information

Below is the link to the electronic supplementary material.

Supplementary file1 Supplementary Figure. 1 End-of-life medical preferences at the time of LVAD implantation and 1 year post-implantation in (A) patients remaining on DT-LVAD and (B) those switched to BTT. BTT, bridge to transplantation; DT, destination therapy; IV, intravenous; LVAD, left ventricular assist device. Supplementary Figure. 2 Life wishes at the time of LVAD implantation and 1 year post-implantation in (A) patients remaining on DT-LVAD and (B) those switched to BTT. BTT, bridge to transplantation; DT, destination therapy; LVAD, left ventricular assist device (PPTX 128 KB)

## Data Availability

The datasets generated and/or analyzed during the current study are available from the corresponding author on reasonable request.
