# Supplementary material for: End-of-life decision-making and changing preferences in patients with a left ventricular assist device for destination therapy: insights from advance directives in Japan
Source: J Artif Organs. 2025 Jul 22;28(4):554–61. doi: 10.1007/s10047-025-01519-6 (PMC12549418; doi:10.1007/s10047-025-01519-6)
Supplement: Supplementary file 1 — Supplementary file1 Supplementary Figure. 1 End-of-life medical preferences at the time of LVAD implantation and 1 year post-implantation in (A) patients remaining on DT-LVAD and (B) those switched to BTT. BTT, bridge to transplantation; DT, destination therapy; IV, intravenous; LVAD, left ventricular assist device. Supplementary Figure. 2 Life wishes at the time of LVAD implantation and 1 year post-implantation in (A) patients remaining on DT-LVAD and (B) those switched to BTT. BTT, bridge to transplantation; DT, destination therapy; LVAD, left ventricular assist device (PPTX 128 KB) [file 10047_2025_1519_MOESM1_ESM.pptx]

## Slide 1
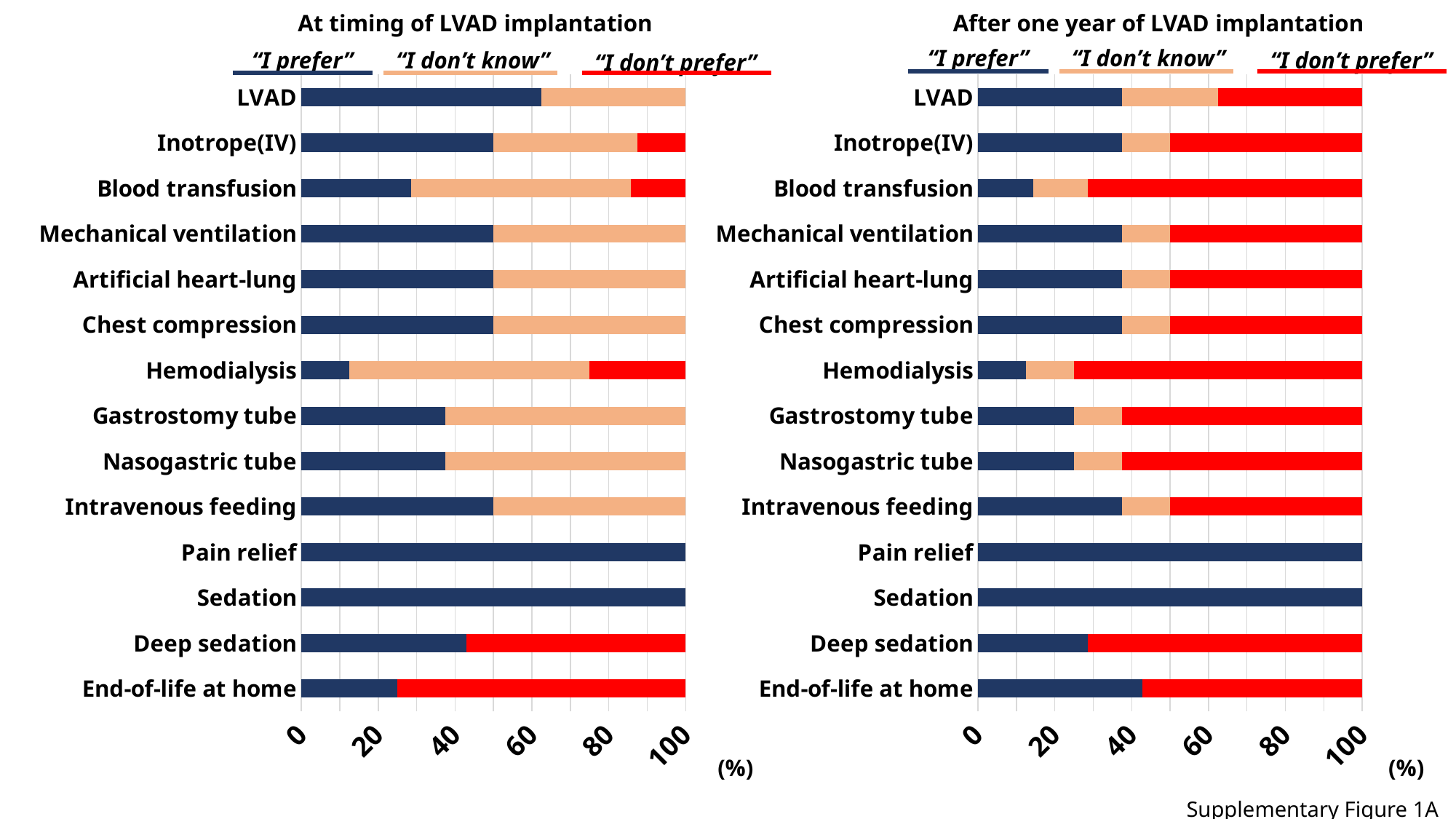

At timing of LVAD implantation
After one year of LVAD implantation
“I prefer”
“I don’t know”
“I prefer”
“I don’t know”
“I don’t prefer”
“I don’t prefer”
### Chart
| Category | 1 | 2 | 3 |
|---|---|---|---|
| End-of-life at home | 25.0 | 75.0 | 0.0 |
| Deep sedation | 42.857142857142854 | 57.14285714285714 | 0.0 |
| Sedation | 100.0 | 0.0 | 0.0 |
| Pain relief | 100.0 | 0.0 | 0.0 |
| Intravenous feeding | 50.0 | 50.0 | 0.0 |
| Nasogastric tube | 37.5 | 62.5 | 0.0 |
| Gastrostomy tube | 37.5 | 62.5 | 0.0 |
| Hemodialysis | 12.5 | 62.5 | 25.0 |
| Chest compression | 50.0 | 50.0 | 0.0 |
| Artificial heart-lung | 50.0 | 50.0 | 0.0 |
| Mechanical ventilation | 50.0 | 50.0 | 0.0 |
| Blood transfusion | 28.57142857142857 | 57.14285714285714 | 14.285714285714285 |
| Inotrope(IV) | 50.0 | 37.5 | 12.5 |
| LVAD | 62.5 | 37.5 | 0.0 |
### Chart
| Category | 1 | 2 | 3 |
|---|---|---|---|
| End-of-life at home | 42.857142857142854 | 57.14285714285714 | 0.0 |
| Deep sedation | 28.57142857142857 | 71.42857142857143 | 0.0 |
| Sedation | 100.0 | 0.0 | 0.0 |
| Pain relief | 100.0 | 0.0 | 0.0 |
| Intravenous feeding | 37.5 | 12.5 | 50.0 |
| Nasogastric tube | 25.0 | 12.5 | 62.5 |
| Gastrostomy tube | 25.0 | 12.5 | 62.5 |
| Hemodialysis | 12.5 | 12.5 | 75.0 |
| Chest compression | 37.5 | 12.5 | 50.0 |
| Artificial heart-lung | 37.5 | 12.5 | 50.0 |
| Mechanical ventilation | 37.5 | 12.5 | 50.0 |
| Blood transfusion | 14.285714285714285 | 14.285714285714285 | 71.42857142857143 |
| Inotrope(IV) | 37.5 | 12.5 | 50.0 |
| LVAD | 37.5 | 25.0 | 37.5 |(%)
(%)
Supplementary Figure 1A

## Slide 2
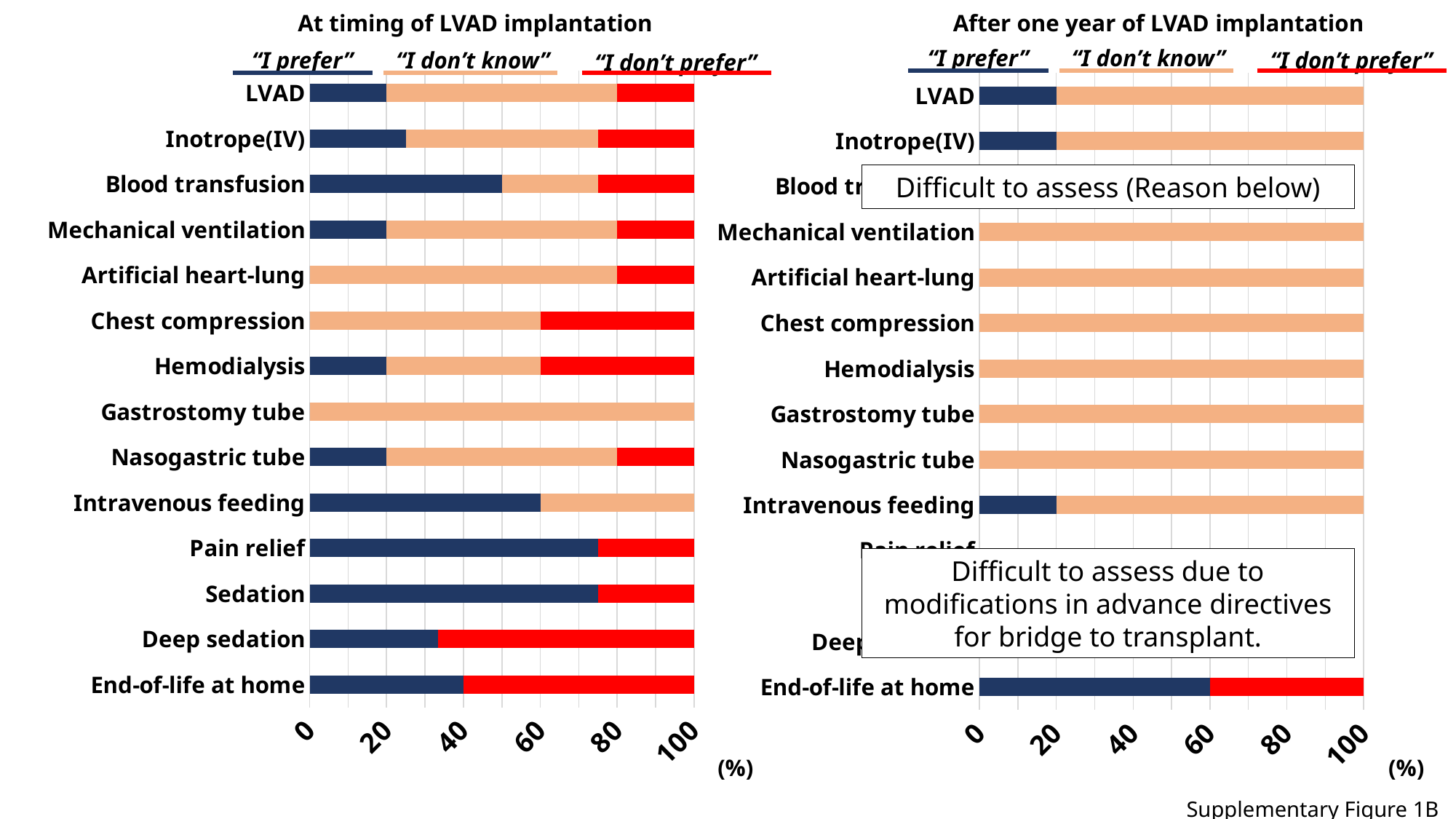

At timing of LVAD implantation
After one year of LVAD implantation
“I prefer”
“I don’t know”
“I prefer”
“I don’t know”
“I don’t prefer”
“I don’t prefer”
### Chart
| Category | 1 | 2 | 3 |
|---|---|---|---|
| End-of-life at home | 40.0 | 60.0 | 0.0 |
| Deep sedation | 33.33333333333333 | 66.66666666666666 | 0.0 |
| Sedation | 75.0 | 25.0 | 0.0 |
| Pain relief | 75.0 | 25.0 | 0.0 |
| Intravenous feeding | 60.0 | 40.0 | 0.0 |
| Nasogastric tube | 20.0 | 60.0 | 20.0 |
| Gastrostomy tube | 0.0 | 100.0 | 0.0 |
| Hemodialysis | 20.0 | 40.0 | 40.0 |
| Chest compression | 0.0 | 60.0 | 40.0 |
| Artificial heart-lung | 0.0 | 80.0 | 20.0 |
| Mechanical ventilation | 20.0 | 60.0 | 20.0 |
| Blood transfusion | 50.0 | 25.0 | 25.0 |
| Inotrope(IV) | 25.0 | 50.0 | 25.0 |
| LVAD | 20.0 | 60.0 | 20.0 |
### Chart
| Category | 1 | 2 | 3 |
|---|---|---|---|
| End-of-life at home | 60.0 | 40.0 | 0.0 |
| Deep sedation | 0.0 | 0.0 | 0.0 |
| Sedation | None | None | 0.0 |
| Pain relief | None | None | 0.0 |
| Intravenous feeding | 20.0 | 80.0 | 0.0 |
| Nasogastric tube | 0.0 | 100.0 | 0.0 |
| Gastrostomy tube | 0.0 | 100.0 | 0.0 |
| Hemodialysis | 0.0 | 100.0 | 0.0 |
| Chest compression | 0.0 | 100.0 | 0.0 |
| Artificial heart-lung | 0.0 | 100.0 | 0.0 |
| Mechanical ventilation | 0.0 | 100.0 | 0.0 |
| Blood transfusion | 0.0 | 0.0 | 0.0 |
| Inotrope(IV) | 20.0 | 80.0 | 0.0 |
| LVAD | 20.0 | 80.0 | 0.0 |Difficult to assess (Reason below)
Difficult to assess due to modifications in advance directives for bridge to transplant.
(%)
(%)
Supplementary Figure 1B

## Slide 3
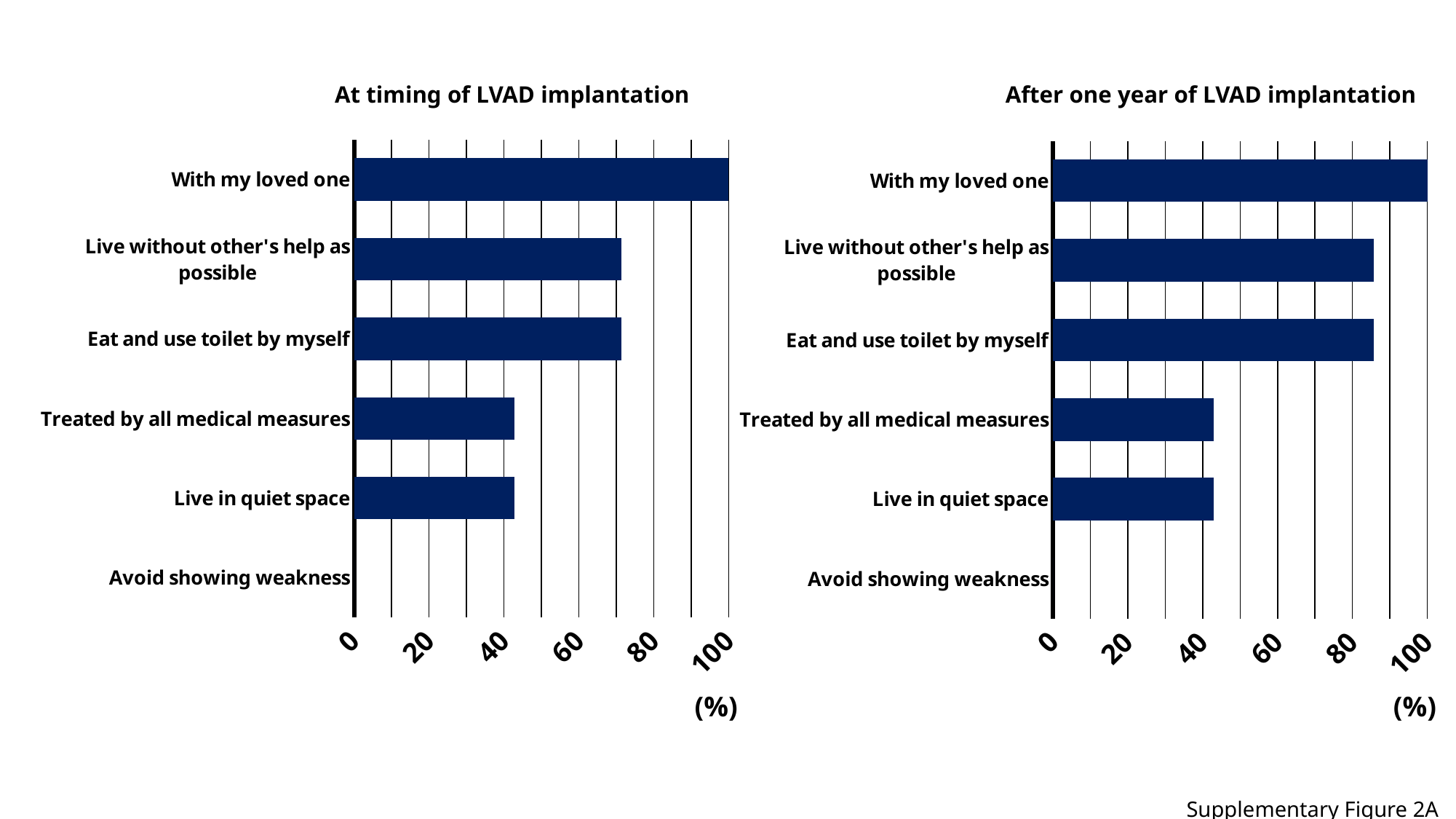

At timing of LVAD implantation
After one year of LVAD implantation
### Chart
| Category | |
|---|---|
| Avoid showing weakness | 0.0 |
| Live in quiet space | 42.857142857142854 |
| Treated by all medical measures | 42.857142857142854 |
| Eat and use toilet by myself | 71.42857142857143 |
| Live without other's help as possible | 71.42857142857143 |
| With my loved one | 100.0 |
### Chart
| Category | |
|---|---|
| Avoid showing weakness | 0.0 |
| Live in quiet space | 42.857142857142854 |
| Treated by all medical measures | 42.857142857142854 |
| Eat and use toilet by myself | 85.71428571428571 |
| Live without other's help as possible | 85.71428571428571 |
| With my loved one | 100.0 |(%)
(%)
Supplementary Figure 2A

## Slide 4
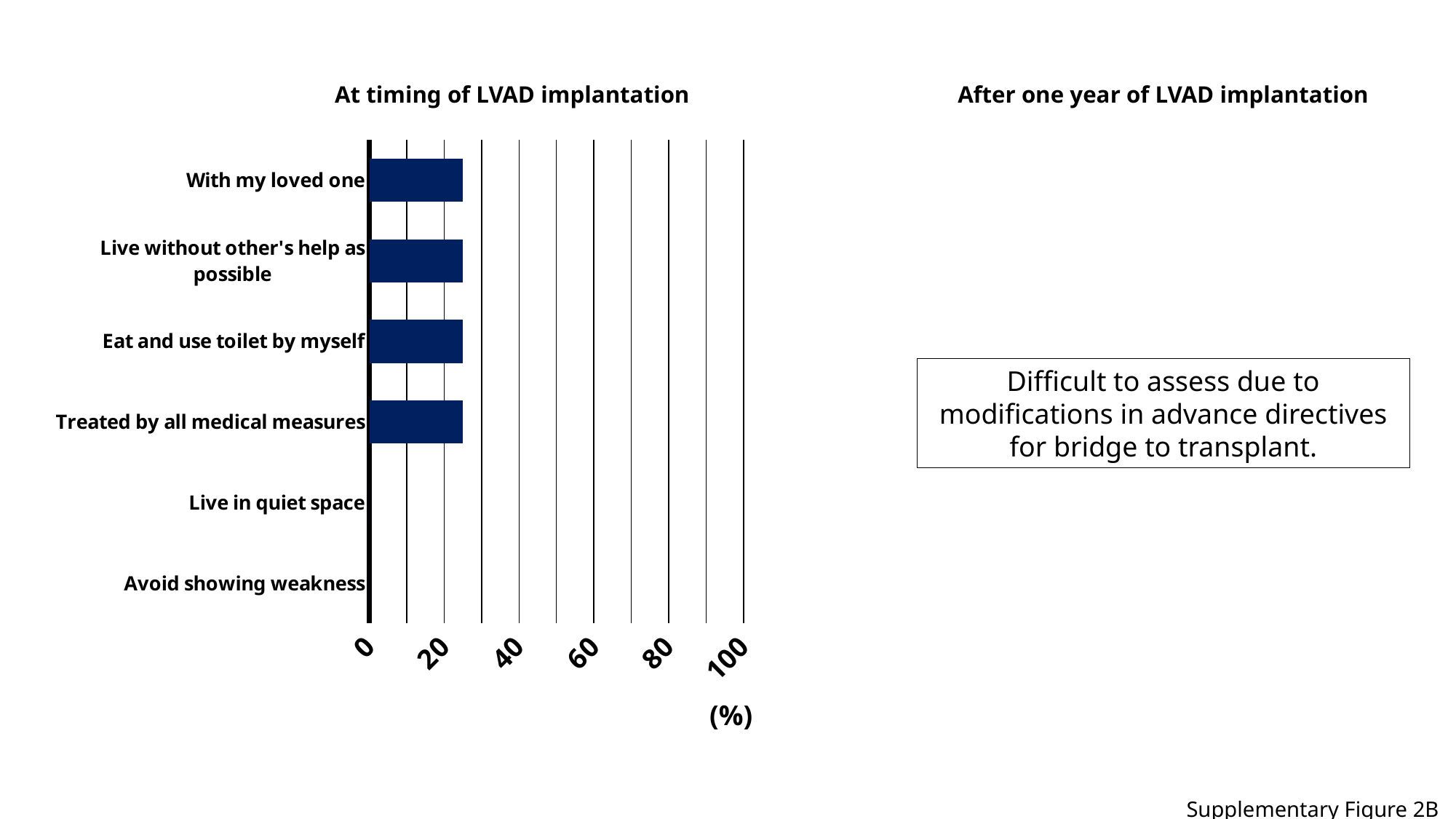

At timing of LVAD implantation
After one year of LVAD implantation
### Chart
| Category | |
|---|---|
| Avoid showing weakness | 0.0 |
| Live in quiet space | 0.0 |
| Treated by all medical measures | 25.0 |
| Eat and use toilet by myself | 25.0 |
| Live without other's help as possible | 25.0 |
| With my loved one | 25.0 |Difficult to assess due to modifications in advance directives for bridge to transplant.
(%)
Supplementary Figure 2B
